# Elevated p53 expression levels correlate with tumor progression and poor prognosis in patients exhibiting esophageal squamous cell carcinoma

**DOI:** 10.3892/ol.2014.2343

**Published:** 2014-07-11

**Authors:** KATE HUANG, LIN CHEN, JILIANG ZHANG, ZHI WU, LINHUA LAN, LU WANG, BIN LU, YONGZHANG LIU

**Affiliations:** 1 Department of Pathology, The First Affiliated Hospital of Wenzhou Medical University, Wenzhou, Zhejiang 325035, P.R. China; 2 Department of Biochemistry and Molecular Biology, Attardi Institute of Mitochondrial Biomedicine, Zhejiang Provincial Key Laboratory of Medical Genetics, School of Laboratory Medicine and Life Sciences, Wenzhou Medical University, Wenzhou, Zhejiang 325035, P.R. China

**Keywords:** esophageal squamous cell carcinoma, p53, immunohistochemistry, western blot, prognosis

## Abstract

Esophageal squamous cell carcinoma (ESCC) is the most common histological subtype of esophageal cancer and one of the most aggressive types of malignancy, with a high rate of mortality. Early diagnosis and treatment may improve the prognosis of ESCC and, thus, survival rates. As a significant tumor suppressor, p53 is closely associated with apoptosis and the differentiation of cancer cells. The present study evaluated the expression levels of the p53 protein and the clinical significance in patients presenting with ESCC. The p53 protein expression level of 64 paired ESCC and tumor-adjacent normal tissues was evaluated using western blot analysis. In addition, immunohistochemistry (IHC) was performed to detect the p53 expression level in specimens from 118 paraffin-embedded cancerous tissues. The correlation of the p53 expression level with the clinicopathological parameters and prognosis of the ESCC patients was also analyzed. The p53 protein was identified to be highly expressed in the ESCC tissue, with western blot analysis demonstrating that the expression level of p53 in the cancerous tissue was 1.89 times that of the tumor-adjacent normal tissue (P<0.001); furthermore, IHC indicated that there was a marked positive expression of p53 in the ESCC tissue (49.15%). The expression level of p53 protein was identified to be significantly correlated with the tumor grade (P<0.001), N stage (P=0.010). Additionally, the higher level of p53 expression was found to be associated with a poor survival rate in the ESCC patients (P=0.0404). The univariate analysis showed that the survival time of patients was significantly correlated with the T stage (RR=3.886, P<0.001), N stage (lymph node metastasis; RR=3.620, P<0.001) and TNM stage (RR=3.576, P<0.001). Furthermore, the multivariate analysis revealed that the T stage (RR=3.988, P<0.001) and N stage (RR=4.240, P=0.004) significantly influenced the overall survival of the ESCC patients.

## Introduction

Human esophageal squamous cell carcinoma (ESCC) is one of the most aggressive types of cancer and is ranked as the sixth most frequent cause of cancer-associated mortality in the world, with a high incidence in northern China, South Africa, Turkey and Iran (1–3). Furthermore, ESCC constitutes 7% of all gastrointestinal cancers and is the predominant histological subtype of esophageal cancer, comprising ~70% of cases worldwide. Recently, progress in early diagnosis, surgery, and chemo- and radiotherapy has led to an increase in the eight-year overall survival rate of ESCC patients, however, improving the prognosis of ESCC patients remains a challenge.

Tumor suppressor p53, encoded by the *p53* gene located at chromosome 17q13.1, is highly associated with a poor prognosis in human cancers (4,5). It is well known that the p53 protein may induce cell apoptosis and regulate cell proliferation. Mutation of the *p53* gene results in the loss of its ability to induce cell death, which leads to uncontrolled cell growth, thus, promoting tumorigenesis (6,7).

In the present study, the overexpression of p53 in the nucleus of the ESCC patient tissues was examined via tissue microarray (TMA), which incorporated 118 ESCC specimens, as well as using western blotting to analyze 64 samples of freshly frozen tissues from ESCC patients.

The correlation between the p53 protein expression level, and tumor progression and prognosis of ESCC patient was evaluated, which may provide further data for predicting the progression and prognosis in patients with ESCC.

## Patients and methods

### Patients and tissue samples

A total of 64 paired tissue samples, including tumor tissue and the adjacent non-cancerous tissue, were collected from ESCC patients who underwent surgery at the Department of Cardiothoracic Surgery, the First Affiliated Hospital of Wenzhou Medical University (Wenzhou, China) between May 2012 and September 2013. The tissues were immediately frozen in liquid nitrogen following surgery and stored at −80°C until undergoing western blot analysis to detect p53 expression levels. Written informed consent for experimental use of the specimens was obtained from all patients and the study was approved by the Board and Ethics Committee of Wenzhou Medical University (Wenzhou, China). All the patients were clinically and pathologically confirmed to exhibit ESCC, and the tumor tissues were classified according to the American Joint Committee on Cancer/Union Internationale Contre le Cancer and were histologically graded in accordance with the World Health Organization classification (8,9).

### Protein extraction and western blot analysis

The total protein from the 64 paired tissue samples was homogenized using a homogenizer (Polytron PT-MR2100; Kinematica AG, Luzern, Switzerland) in 1.5 ml tissue radio-immunoprecipitation assay lysis buffer (50 mM Tris [pH 7.4], 150 mM NaCl, 1.0% Triton X-100, 1% sodium deoxycholate and 0.1% SDS; Beyotime Institue of Biotechnology, Shanghai, China) containing protease inhibitor cocktail (Roche Applied Science, Indianapolis, IN, USA), 1 mM NaF and 1 mM Na_3_VO_4_. Tissue homogenates were incubated on ice for 15 min, centrifuged (Centrifuge 5417R; Eppendorf, Hauppauge, NY, USA) at 18,000 × g for 20 min at 4°C and the supernatants were collected. The protein concentration was subsequently quantified using a BCA Protein assay kit (Thermo Fisher Scientific, Waltham, MA, USA). A total of 20 mg protein from each sample was separated by 10% SDS-PAGE (Bio-Rad, Hercules, CA, USA) and transferred onto a nitrocellulose membrane (Bio-Rad). Immunoblot analysis was subsequently performed with monoclonal rabbit anti-human p53 (Proteintech Group, Wuhan, China) and monoclonal mouse anti-human actin (Abmart Inc., Shanghai, China) antibodies. The horseradish peroxidase-conjugated secondary antibodies were obtained from Abmart Inc. The signals were visualized using an electrochemiluminescence system (Thermo Fisher Scientific) according to the manufacturer’s instructions and the optical density was quantified using the National Institutes of Health ImageJ software (http://imagej.nih.gov/ij/download/).

### TMA and immunohistochemistry (IHC)

An ESCC TMA, containing a total of 118 formalin-fixed paraffin-embedded tissue samples, was constructed according to a previously described method (10). IHC was also performed according to a previously described method (11). Briefly, the sections were deparaffinized in xylene and rehydrated through a gradient concentration of alcohol. The endogenous peroxidase activity was inactivated, non-specific staining was blocked by 5% normal goat serum and all sections were incubated with anti-p53 antibody (1:100; Abmart Inc.) overnight at 4°C. The slides were incubated with biotin-labeled goat anti-rabbit immunoglobulin G and further incubated with streptavidin peroxidase solution (SABC kit, Boster Biological Technology, Ltd., Wuhan, China). The staining was visualized by reaction with 3, 3′-di-aminobenzidine (Boster Biological Technology, Ltd.) in phosphate-buffered saline [PBS; Dycent Biotech (Shanghai) Co. Ltd., Shanghai, China] with 0.05% H_2_O_2_ for 5 min at room temperature. Control staining was performed by staining the same TMA (duplicate) with PBS rather than anti-p53 and no immunostaining was observed. The slides were counter-stained with hematoxylin, washed in double-distilled H_2_O and mounted with resinous mounting medium. The TMA were scored separately by two pathologists who had no prior knowledge of the clinicopathological status of the specimens on the TMA.

### Assessment of IHC

Histopathological sections were microscopically examined (Nikon ECLIPSE 80i; Nikon Corporation, Tokyo, Japan) and scored by two independent pathologists, who were blinded to the clinical data pertaining to the patients. The IHC staining of mutant (MT)p53 was assessed according to the immune-reactive score (IRS) as described previously (12,13) with slight adjustments, which evaluated the percentage of positive cells and the staining intensity. The percentage of positive cells was scored as follows: 1, ≤10% positive cells; 2, 11–49%; 3, 50–79%; and 4, ≥80% (14). The staining intensity was graded as 0, negative; 1, weak; 2, moderate; and 3, strong. The two scores were multiplied and the IRS (a value between 0 and 12) was determined as low or high, which corresponded to IRS values of ≤6 and >6, respectively.

### Statistical analysis

The optical density of the western blot signals was quantified using the National Institutes of Health ImageJ software and all statistical analyses were carried out using the SPSS 13.0 statistical software package (SPSS Inc., Chicago, IL, USA). The expression level of p53 was quantified relative to β-actin and the differences between the cancer tissues and adjacent normal tissues in p53 protein expression levels were compared using Student’s t-test. The χ^2^ test was performed to evaluate the correlation between the clinicopathological features of the patients and the p53 expression level, which was observed by IHC. Kaplan-Meier survival analysis was used to evaluate the patient prognosis and the eight-year survival rate of the ESCC patients was obtained using the life table method. A univariate analysis was plotted using the Kaplan-Meier method and Cox regression analysis was used to evaluate the correlation between risk of ESCC and clinicopathological parameters, including p53 expression. P≤0.05 was considered to indicate a statistically significant difference.

## Results

### Increased level of p53 expression was observed in ESCC tissues when compared with paired non-neoplastic tissues

**Figure 1:**
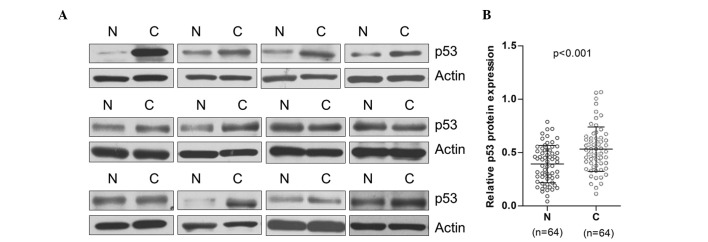
p53 protein expression in esophageal squamous cell carcinoma (ESCC) patient tissues. (A) Western blot analysis of p53 protein expression in ESCC patient tumor tissue (denoted as C) and matched tumor-adjacent non-neoplastic tissue (denoted as N). (B) The quantitative results of western blot analysis demonstrated that p53 expression was increased in the tumor tissue when compared with the matched non-neoplastic tissue of the ESCC patients. (n=64, P<0.001).

In the present study, the p53 protein expression level of 64 paired tumor tissues and non-neoplastic tissues was analyzed using western blot. The result demonstrated that the p53 expression level in the ESCC tissues was significantly higher than that in the matched non-neoplastic tissues. The p53 protein in the tumor tissue of the ESCC patients was found to be 1.89 times that of the matched non-neoplastic tissues (n=64, P<0.001; Fig. 1A and B).

### IHC of p53 expression levels in ESCC and matched non-neoplastic tissues

**Figure 2:**
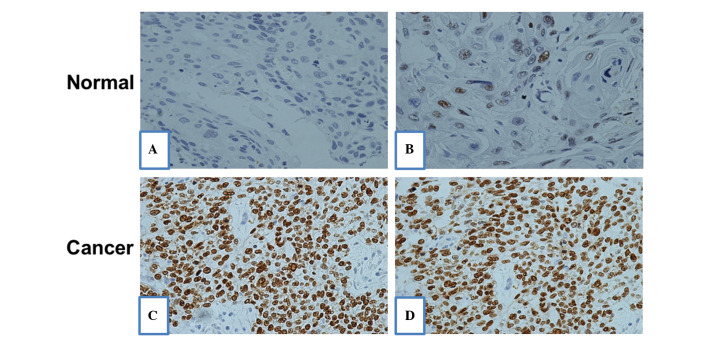
Representative immunohistochemistry for p53 in esophageal squamous cell carcinoma tissues (magnification, ×400). (A and B) Tumor-adjacent non-neoplastic tissue and (C and D) tumor tissue. The p53 protein is predominantly localized in the nucleus of the cancer cells.

IHC of ESCC TMA was conducted to further evaluate the level of p53 protein expression in the ESCC tissues. IHC revealed that the p53 protein was predominantly localized in the nucleus (Fig. 2A) and the expression level of p53 in the ESCC tissue was identified to be significantly higher when compared with that in the adjacent normal tissues (Fig. 2A).

### Association of the p53 protein expression level with clinicopathological features

A total of 118 ESCC patient tissue samples were used to construct the TMA, including 96 males and 22 females (age range, 26–79 years; median, 63 years). The IHC staining for p53 (MTp53) demonstrated low and high levels of p53 expression in 60 (50.8%) and 58 (49.15%) samples, respectively. The level of p53 protein expression was found to correlate with the pathological grade (P<0.001) and N stage (P=0.007), however, not with patient age, gender, history of alcohol consumption and smoking, T stage or TNM stage (Table I).

### Survival analysis

**Figure 3:**
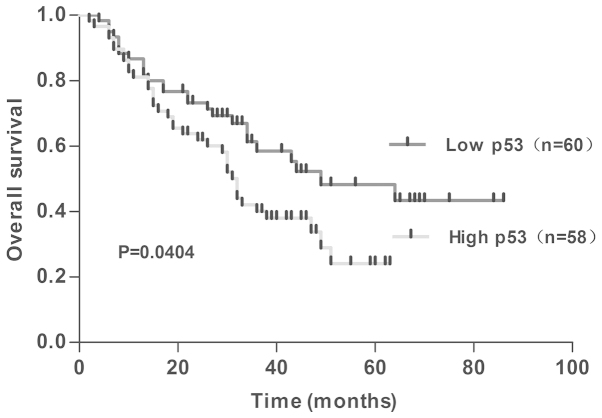
Correlation between p53 protein expression status and prognosis of esophageal squamous cell carcinoma (ESCC) patients. The Kaplan-Meier overall survival curve of ESCC patients (n=118) according to p53 protein expression level demonstrated that ESCC patients exhibiting high levels of p53 protein expression are associated with a poor overall survival. P=0.0404.

The patients with clear follow-up data were used for the survival analysis. Out of 118 patients, there were 58 cases that exhibited a high expression level of p53 (49.15%), and 60 cases (50.85%) that exhibited a low expression level of p53. The eight-year survival rate was 36.21% (21/58), 56.67% (34/60), in the high p53 expression group and low p53 expression group, respectively. Survival curves were obtained using the Kaplan-Meier analysis and the log-rank test was used to compare differences in survival between the two groups. According to the survival analysis, it was found that the eight-year survival rate of the group with low levels of p53 protein expression was higher than the group with high levels of p53 protein expression (P=0.0404; Fig. 3).

### Assessment of ESCC risk factors

The univariate analysis and multivariate analysis were used to evaluate the influence of various parameters on the disease-free survival rate of ESCC patients. The ESCC risk factors, including the p53 expression level, patient age, gender, TNM stage, pathological grade, N stage, T stage, and history of smoking and alcohol consumption were taken into account (Table II). The univariate analysis demonstrated that the survival time of patients was significantly correlated with the T stage (RR=3.886, P<0.001), N stage (RR=3.620, P<0.001) and TNM stage (RR=3.576, P<0.001). Furthermore, multivariate analysis revealed that the T stage (RR=3.988, P<0.001) and N stage (RR=4.240, P=0.004) were significant and independent prognostic factors for ESCC patients.

## Discussion

The human *p53* gene is located at chromosome 17p13.1 and encodes the p53 protein, which is composed of 393 amino acids. The *p53* gene is a member of a highly conserved family that contains at least another two genes, *p63* and *p73*. The wild-type (WT)p53 protein contains 393 amino acids and p53 is a tumor suppressor that has a close association with numerous types of human cancer; the mutation or loss of the *p53* gene can be identified in >50% of all human cancers (15,16). p53 is involved in the regulation of the cell cycle, as well as inducing a variety of activities to maintain the genomic stability, cellular senescence (17,18) and apoptosis (19). Under normal conditions, p53 protein levels are maintained at a very low level unless the cells are activated by signals from DNA damage, as well as certain other cellular stresses (20). The response to DNA damage and cellular stresses is the upregulation of the p53 protein expression level, which leads to cell cycle arrest, DNA repair or apoptosis. Thus, p53 is critical in the inhibition of malignant cancer cell division.

There are two types of p53 proteins, WTp53 and MTp53. WTp53 is a tumor suppressor, which prevents the proliferation of tumor cells; MTp53 causes issues with the regulation of the cell cycle, resulting in uncontrolled cell growth that promotes tumorigenesis. WTp53 has a particularly short half-life and is difficult to detect in normal cells. Conversely, MTp53 is markedly more stable, with a longer half-life, which favors detection by IHC. Previous studies have detected the p53 mutation using IHC and were able to define the tissue via the strong staining of the p53 protein as MTp53 (21–23). Based on this finding, the point mutation in the *p53* gene was associated with p53 protein stabilization. The majority of human cancers may be detected via the upregulation of the p53 protein, including liposarcoma (24), colorectal cancer (25), breast carcinomas (26) and endometrial carcinomas (27). Recently, Zhu *et al* (28) demonstrated that the knock-down of MTp53 using small interfering RNA induced cell cylce arrest and triggered apoptosis in bladder cancer cells.

Chava *et al* (29) performed IHC in archival tissue samples to evaluate the expression levels of fragile histidine triad (FHIT) and the p53 protein. The study indicated that the level of *p53* gene expression was eight times that which was observed in the normal tissues. The results showed that FHIT and *p53* were well correlated with SCC. As the study only involved 23 ESCC samples to perform the IHC, a greater number of samples are required to improve the evaluation of the correlation of the p53 protein with ESCC. In the present study, western blot analysis was performed using tissues, which were snap-frozen in liquid nitrogen and stored at −80°C. The results demonstrated that the expression level of p53 protein in the cancer tissues was 1.89 times than that in the normal tissue. In addition, IHC analysis was conducted and the results demonstrated that the tumor tissues of the ESCC patients exhibited strong p53 protein staining, whereas the matched tumor-adjacent tissues exhibited weak p53 staining. Therefore, the upregulation of p53 in ESCC patient tissues has a significant role in esophageal carcinomaproliferation.

ESCC tumorigenesis is a complex process, which is affected by various factors. The pathogenesis of ESCC remains unclear, and numerous studies indicate that ESCC is associated with multi-factor and multi-gene mutations. However, previous studies have shown that environmental and lifestyle factors, such as smoking, alcohol consumption, lack of fruit and vegetable intake, or an excess of pickled foods are potential factors that may lead to esophageal cancer (29–33). In the present study, the Cox proportional hazards model was used during the statistical analysis, and revealed that the patient age, gender, clinical and pathological stages, and the presence or absence of a history of alcohol and tobacco use did not result in significant differences with regard to an association with prognosis; however, the results of univariate analysis showed that the T stage, N stage and TNM stage were significantly correlated with the prognosis of patient survival. Additionally, multivariate analysis revealed that the T and N stages correlated with ESCC patient survival.

In conclusion, the present results further demonstrated that p53 (MTp53) is overexpressed in the tumor tissue of ESCC patients, which leads to transcriptional regulation dysfunction and uncontrolled cell growth. Therefore, p53 may be used as a specific therapeutic target for the treatment of ESCC and as a biomarker for the diagnosis of ESCC.

## Figures and Tables

**Table I:** Association between p53 expression and various clinicopathological factors of esophageal squamous cell carcinoma patients.

Characteristic	Total, n=118	p53 protein expression		P-value

		Low, n=60	High, n=58	
Gender				0.391
Male	96	47	49	
Female	22	13	9	
Age				0.732
<60	49	24	25	
≥60	69	36	33	
Smoker				0.721
Yes	63	33	30	
No	55	27	28	
Alcohol consumer				0.732
Yes	69	36	33	
No	49	24	25	
Pathological grade				<0.001^b^
G1	37	29	8	
G2	56	27	29	
G3	25	4	21	
T stage^a^				0.062
T1	23	17	6	
T2	36	14	22	
T3	56	28	28	
T4	3	1	2	
N stage^a^				0.010^b^
N0	67	41	26	
N≥1	51	19	32	

a According to the TNM Classification of Malignant Tumours.

b P<0.05 was considered to indicate a statistically significant difference. The χ^2^ test was used to analyze the association between p53 expression levels and clinicopathological characteristics.

T, tumor; N, necrosis; M, metastasis.

**Table II:** Univariate analysis and multivariate analysis identifies the factors that influence the overall survival rate of esophageal squamous cell carcinoma patients.

	Univariate analysis			Multivariate analysis		
		
Variable	RR	95% CI	P-value	RR	95% CI	P-value
p53	1.646	0.987–2.745	0.056	1.282	0.736–2.233	0.381
Age	1.007	0.979–1.035	0.652	1.012	0.984–1.040	0.408
Gender	1.282	0.651–2.523	0.472	1.160	0.501–2.685	0.728
Smoker	1.095	0.661–1.811	0.725	1.755	0.756–4.077	0.191
Alcohol consumer	0.948	0.570–1.577	0.948	0.531	0.224–1.259	0.151
Pathological grade	1.342	0.749–2.404	0.323	1.057	0.571–1.955	0.860
T stage^a^	3.886	2.256–6.696	<0.001	3.988	1.969–8.077	<0.001
N stage^a^	3.620	2.149–6.099	<0.001	4.240	1.580–11.378	0.004
TNM stage^a^	3.576	2.144–5.963	<0.001	0.596	0.207–1.716	0.381

a According to the TNM Classification of Malignant Tumours. The Cox proportional hazards model was used to identify the factors that had a significant influence on overall survival. P<0.05 was considered to indicate a statistically significant difference.

RR, relative risk; CI, confidence interval; T, tumor; N, necrosis; M, metastasis.
